# Investigating diet to control asparagine uptake as an adjunct to asparaginase treatment

**DOI:** 10.3389/fonc.2025.1634113

**Published:** 2026-01-30

**Authors:** Zara Forbrigger, Tamara MacDonald, Ketan Kulkarni, Andrew W. Stadnyk

**Affiliations:** 1 Department of Pathology, Dalhousie University, Halifax, NS, Canada; 2 Division of Hematology & Oncology, IWK Health, Halifax, NS, Canada; 3 Department of Pediatrics, Dalhousie University, Halifax, NS, Canada; 4 Department of Microbiology & Immunology, Dalhousie University, Halifax, NS, Canada

**Keywords:** leukemia, asparaginase, diet, asparagine, metabolome, microbiome, cancer

## Abstract

Ongoing refinements of multidrug regimens, and particularly the addition of L-asparaginase, resulted in an immediate gain in survival for pediatric acute lymphoblastic leukemia patients. Yet L-asparaginase has substantial side effects which may require dose reductions or delays in subsequent doses. There are at least 3 possible sources of L-asparagine to consider when balancing blood levels with asparaginase dosing, diet, cell synthesis and bacterial synthesis. To date, there is one precedent, in mice, in which blood L-asparagine levels are reduced as a consequence of reducing consumed levels. We build on that approach in experiments aimed at testing whether long-term dietary restriction of L-asparagine and possibly gut bacteria can impact blood levels. In our experiment, 2 groups of mice received food pellets with either 4% or 0% L-asparagine. Blood and fecal metabolites and fecal bacteria were sampled over 72 days. After this accommodation period, all mice continued their diet and received a single injection of pegylated *E. coli* recombinant L-asparaginase. Samples for bacteria and metabolites were collected 4 and 5 days later, respectively. Neither diet had adverse effects on the general health of the mice nor did diet alone change blood L-asparagine levels. Both diets led to changes in gut bacteria. L-asparaginase depleted blood L-asparagine in mice consuming either diet. Bacteria identified in fecal pellets revealed that the microbiomes of mice in the 2 cages were different (cage effect) and remained different although metagenomic analyses of day 72 feces indicated there were no diet-dependent differences in bacterial asparaginase or asparagine synthetase. These outcomes indicate that mice recover from any short-term down regulation of blood L-asparagine due to diet and consequently the metabolic controls become complex, and the gut microbes seem to not be a great influence. Further research should include approaches to determine the source of L-asparagine in the blood while ingesting diets with no/low or high amounts of L-asparagine.

## 1 Introduction

Acute lymphoblastic leukemia (ALL) is the most common childhood cancer accounting for ~25% of all cancer diagnoses (1). Adult ALL is much less common, accounting for only 0.3% of all new cancer cases (2). In ALL, lymphocytes cease development as lymphoblasts and begin to rapidly proliferate. These lymphoblasts overwhelm the patient’s bone marrow, crowding the remaining healthy cells and lead to patients experiencing anemia, infections and thrombocytopenia (3). Treatment for pediatric ALL consists of 2–4 years of chemotherapy depending on risk and the patients’ sex, while the treatment length for adult patients is between 1.5–3 years (4). During this period patients will receive multiple therapeutic agents.

One therapeutic agent in the cocktail of drugs used to treat pediatric ALL is L-asparginase (lately pegaspargase, a pegylated bacterial L-asparaginase (P-ASP)). Asparaginase works primarily by converting L-asparagine (Asn) to aspartic acid and secondarily by catabolizing glutamine into glutamate. Most leukemic cells are unable to synthesize Asn and are entirely reliant on exogenous sources. P-ASP depletes plasma Asn consequently killing leukemic but not healthy cells. Adding L-P-ASP to the ALL treatment strategy lead to an increased overall 3-year survival rate of 73% in comparison to 58% from non-pediatric protocols (5). A study expanding the use of P-ASP beyond children reported that P-ASP can be safely tolerated in older adolescents and young adults <40 years of age (6). P-ASP, therefore, is beneficial in the combination chemotherapy of both pediatric and older ALL patients.

Despite widening use of P-ASP there are concerning adverse events to using the drug. Hypersensitivity reactions to the bacterial protein or to PEG, hyperglycemia, pancreatitis, central nervous system thrombosis, coagulopathy, hyperbilirubinemia, and elevated transaminases are reported side effects (7). High rates of adverse events during use of P-ASP are an indicator that treatment is still sub-optimal and methods to reduce the frequency of adverse events should be explored.

Considering the mode of action of P-ASP another means to possibly reduce Asn levels may be through controlling dietary sources, but there is little history of clinical research into this possibility. For reasons related to liver toxicity in leukemic patients and before L-asparaginase was in use, Halikowski and co-workers trialed a low-protein low-purine diet in 13 pediatric ALL patients and all patients showed an increase in treatment response (8). One factor that needs to be taken into consideration regarding how diet may influence blood levels is the gut bacterial microbiome. Indeed, a study by Visconti et al. in 2019 found that 46% of blood metabolites are associated with the microbiome, highlighting the dynamic relationship between the blood and the gut microbiome (9). Regarding the gut bacteria and pediatric ALL, a study by Dunn et al. (2020) found a relationship between serum P-ASP activity and gut microbial communities (10). Lastly, there is a precedent, using mice, that reported a low Asn diet for a week resulted in low blood levels, and that the low blood Asn impacted the metastatic capacity of a breast cancer cell line (11). In our study, we further address the possibility of regulating blood Asn through diet using a model in which mice were fed pellets with high or low Asn content, and report the blood and fecal metabolomes and gut bacterial microbiome post-diet and post-P-ASP.

## 2 Materials and methods

### 2.1 Animals and sample collection

Eleven 5-week-old C57BL/6 female mice (Charles River, Saint-Constant, QC) were divided into 3 cages by staff at IWK Health who were unrelated to the study. Females were chosen to minimize aggression in the cages as a variable, especially following when mice were separated from the cage during collection of fecal pellets, but in addition, the precedent for a diet/blood Asn relationship by Knott et al., 2018 was conducted using female mice (11). The mice acclimated to the facility for 2 weeks. Ten of the 7-week-old mice were combined into 2 cages of 5 mice each. All handling protocols/treatments were approved by the institutional animals in research ethics committee, who apply the guidelines of the Canadian Council on Animal Care. Saphenous blood (50µL) and fecal samples (~50mg for microbiome and ~100mg for metabolome) were collected from mice prior to and on fixed days during the study period. Fecal samples were collected by placing a mouse into a cage for up to 1 hour ensuring the fresh sample could be related back to each specific mouse.

Mice baseline weights were collected prior to starting their diets. Mice were weighed once a week to track any weight changes during adaptation to the diets. Mice were provided exclusively with either an Asn-rich (4% Asn, DYET# 519593, Dyets Inc., Bethlehem, PA) or an Asn-depleted diet (0%, DYET# 519592) (11) on day 0 (**Supplementary Tables 1** and **2**). From each mouse, on day 35 a 50µl blood sample with heparin anticoagulant was collected, and on day 36 a fresh fecal pellet was collected. On day 72 of diet, blood and fecal samples were again collected, then mice were injected intraperitoneally with 200 IU/kg of P-ASP (ONCASPAR™, Servier Canada Inc., Laval, QC). The dose of P-ASP was a conservative choice based on multiple reports using P-ASP (12–14). Two days post-P-ASP injection (day 74), samples for metabolome analyses were collected. Four- and 5-days post-P-ASP injection microbiome and metabolome samples were collected, respectively.

### 2.2 Sample Preparation for Metabolome and bacterial analyses

To prevent P-ASP from depleting any Asn remaining following blood collection, all samples (whether mice received P-ASP or not) were quenched using a fixed volume of 20% formic acid in ultrapure water, following the methodology published by Horvath et al., 2019 (15). One unintended outcome of this quenching was that both plasma and cell metabolites were isolated. A solution of methanol containing 1% formic acid was added to each sample after the quenching, to precipitate protein. After vortex mixing and centrifugation (17000 x g), samples were evaporated to dryness using a Savant ISS110 SpeedVac Concentrator. The samples were stored at -80 °C until the end of the experiment when all samples were prepared as a single batch for metabolome analysis. Preparation for analysis consisted of adding 1% formic acid in ultrapure water to each sample, vortex mixing, adding acetonitrile with 1% formic acid, vortex mixing and centrifugation (17000 x g).

For fecal metabolomic analyses, 100mg of a fresh fecal pellet had 1:3 ratio (w/v) of methanol added. The samples were homogenized until the feces was in suspension. Samples were then stored at -20 °C for 1 hour. After an hour, the samples were centrifuged and the methanol fraction was collected. An internally labelled isotope standard was then added (5 μL) to each sample. All samples were prepared in a single batch. Fecal samples for bacterial analyses were stored at -80 °C until all sample collection was complete, then the feces were thawed and DNA extracted using the Norgen Biotek Corp. (Thorold, ON) Fecal DNA Isolation Kit.

### 2.3 Sample analyses

Samples for metabolomics were sent to the Biological Mass Spectrometry Centralized Operation of Research Equipment and Support facility at the Faculty of Medicine, Dalhousie University, for analysis using LC-MS/MS. The data were sent to staff at the National Research Council of Canada in Halifax for analysis. Samples for bacteria identification were sent to the Integrated Microbiome Resource, Dalhousie University, for 16S amplification and sequencing as well as metagenome sequencing and preliminary data analysis based off of QIIME2 using Amplicon Sequence Variants (16).

### 2.4 Statistical analysis

Independent t-tests were run on the weight change from starting weights for each week of the study. Comparisons of metabolome data between groups were tested using ANCOVA, with a p value of <0.05 indicating significance. For day 35 and 72 samples, day 0 samples were used for the covariate. For day 77 samples, day 72 samples were used as the covariate. Microbiome analysis was performed using STAMP (17). Metaboanalyst 5.0 GlobalTest with a p-value <0.05 was used to create PCA plots and identify affected pathways (18).

## 3 Results

**Figure 1:**
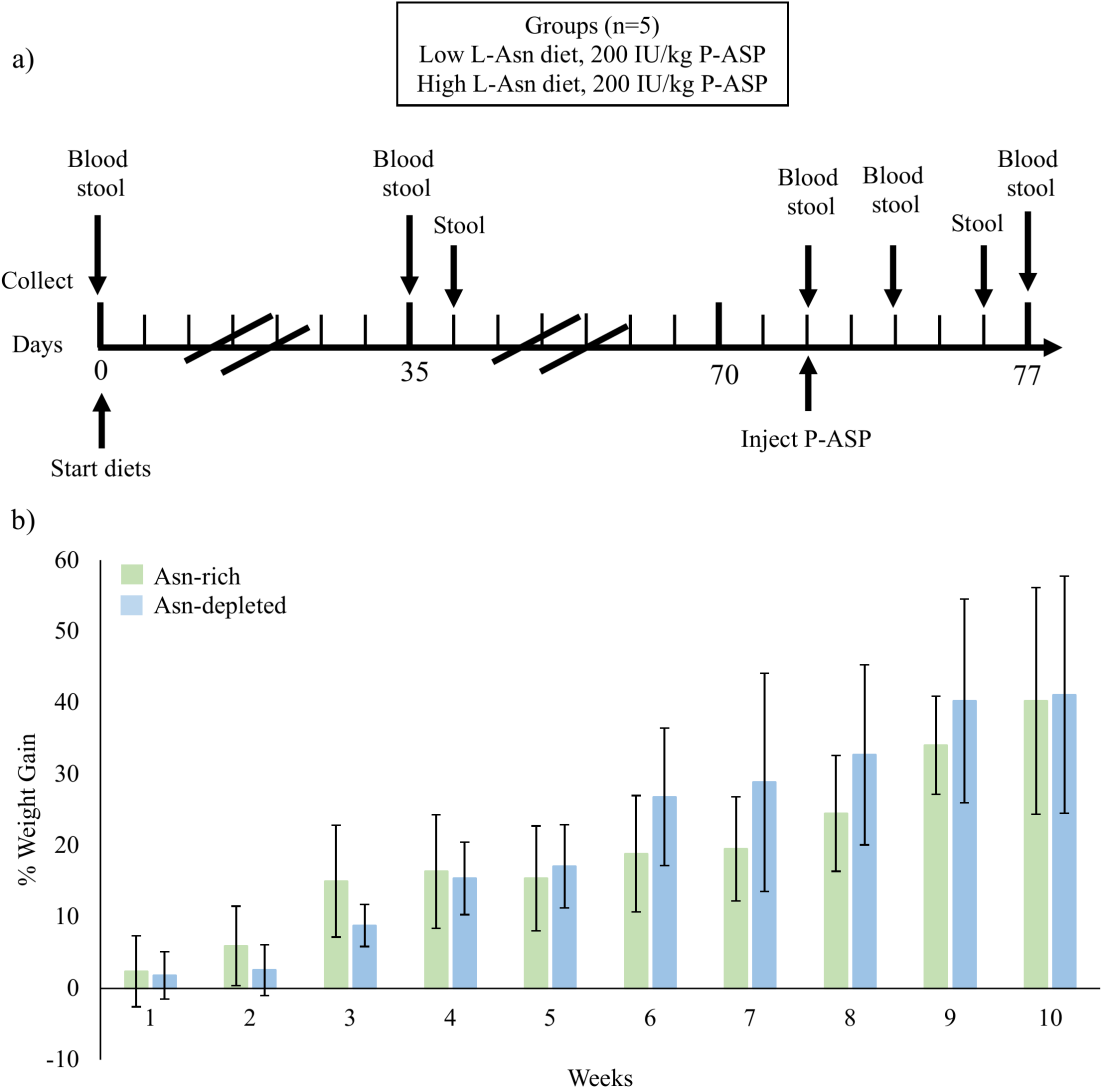
The treatment regimen and mice weight as an indicator of health status. **(a)** Mice were fed a diet of low Asn (0%) or high Asn (4%). Blood and fecal samples were collected from mice throughout the study for metabolome and microbiome analysis. On day 72 mice were injected with P-ASP and on day 77 all mice were euthanized after the final sample collection. **(b)** Average weight of mice on the Asn rich and depleted diets across the study period. Error bars represent standard deviation. Mice were weighed weekly starting on the day they commenced their diets. Independent t-tests indicate no significant difference in weight between treatment groups by week 10 (a p-value < 0.05 indicates significance).

Mice were separated to receive diets with 1 of 2 concentrations of Asn over a period of 72 days (adaptation period) before receiving an injection of P-ASP, then continued their respective diet for 5 more days (**Figure 1a**). Both groups of mice gained a similar amount of weight during the study (p = 0.7401, **Figure 1b**). Mice weighed an average of 24.8g for the Asn-rich diet and 26.9g for the Asn-depleted diet, weights within or above the standard deviation of Jackson laboratories weight guide for mice of the same age (23.5 ± 2.3g) (19). This pattern of weight gain, combined with regular observations of the mice, indicates there were no apparent ill effects of acutely changing their diet, nor due to the diet for the long term. On day 41 of the study, mouse A1 escaped while being handled and had to be euthanized and consequently data is missing from day 72 to 77. Mouse A2 received roughly half the amount of P-ASP during the intraperitoneal injection as the other mice and consequently days 74, 76, and 77 samples were excluded from analysis.

### 3.1 Metabolomics

**Figure 2:**
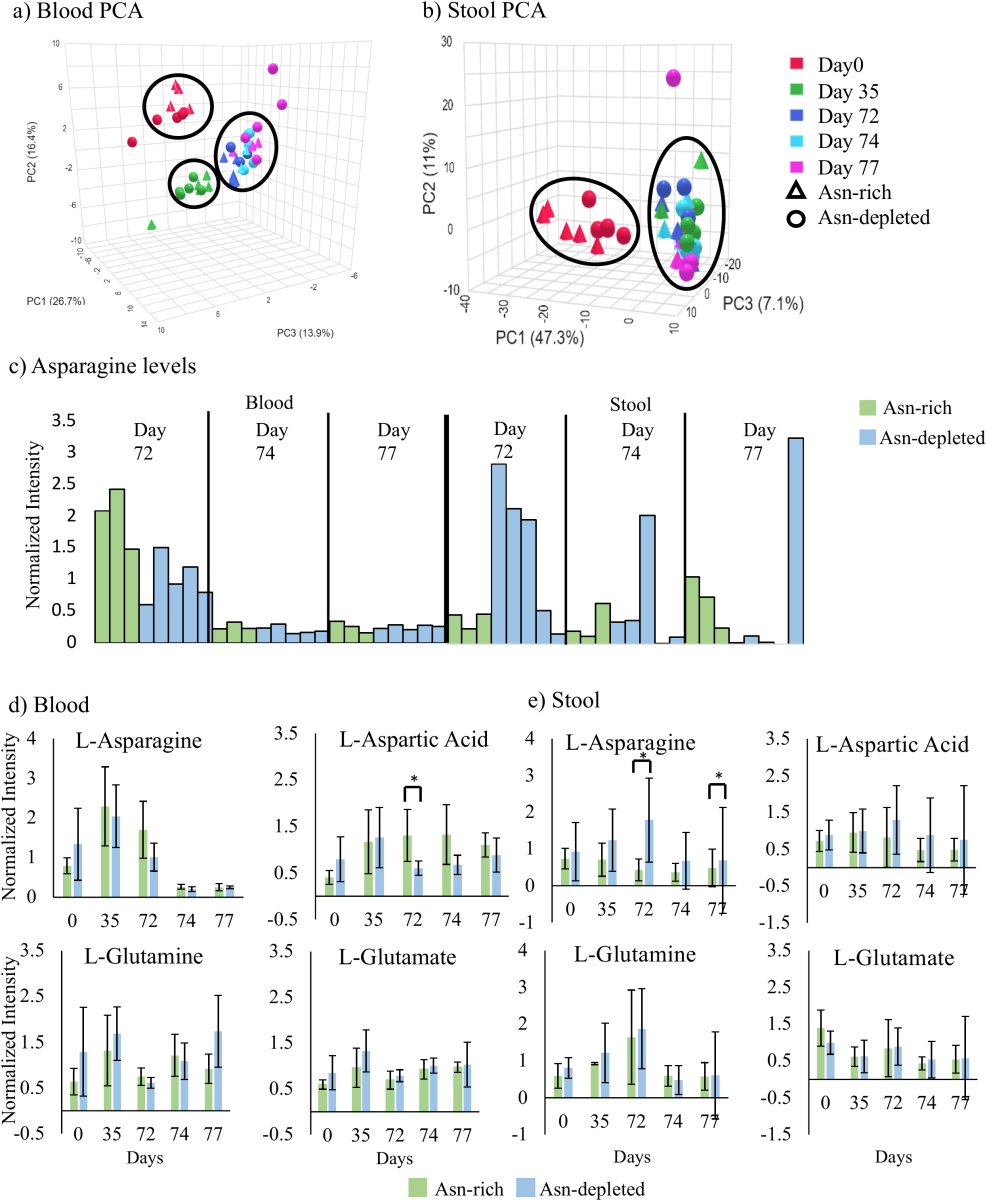
Blood and fecal metabolomics over the diet and treatment time course. LC-MS/MS metabolome results, error bars represent standard deviation. Normalized intensity represents the abundance of each metabolite after correction. **(a)** Blood metabolome PCA plot for samples collected throughout the study. Day 72, 74, and 77 samples clustered together. Day 0 samples and day 35 samples clustered separately. The Asn rich and depleted diet did not cluster separately. **(b)** Fecal metabolome PCA plot for all samples collected throughout the study. Day 0 samples clustered separately. Day 35, 72, 74, and 77 samples clustered together. The samples did not cluster separately based on the treatment group. **(c)** blood and fecal Asn levels for each individual mouse prior to P-ASP injection (day 72) and post P-ASP injection (days 74 and 77). Bars are color coded based on the treatment group. Blood Asn levels were depleted for all mice, up to 5 days post P-ASP. Fecal Asn levels in the Asn depleted diet were lowered for all but one mouse that had a sharp increase in fecal Asn. **(d)** Average blood metabolite levels for mice treated on the Asn rich or depleted diets across the study period. Significance is shown for between groups differences, p value <0.05 was taken as significant. Aspartic acid levels were significantly different between groups on day 72. **(e)** Average fecal metabolite levels for mice treated on the Asn rich or depleted diets across the study period. Significance is shown for between groups differences, p value <0.05 was taken as significant. Fecal Asn levels were significantly different between groups after 72 days on diet and 5 days post P-ASP.

Blood metabolome PCA plots on the PC1 vs PC2 axis showed two distinct clusters (**Figure 2a**). One cluster contained the day 0 samples and the other included most other samples. This pattern indicated that both groups of mice experienced similar changes from a common pattern prior to starting the diets. PCA plots of fecal metabolome samples on the PC1 vs PC2 axis also showed two distinct clusters (**Figure 2b**). Like the blood result, the first cluster contained day 0 samples and the second included days 72, 74 and 77 samples. These outcomes indicate a similar metabolome shift between diet groups by the first time point since starting the diet which then remained relatively constant.

Following the P-ASP injection, blood Asn levels were significantly depleted 2 and 5 day samples (days 74 and 77, respectfully) in both the Asn-rich and Asn-depleted diet groups (**Figure 2c**). On day 77, fecal Asn levels were depleted for all but one mouse in the Asn-depleted diet group, which showed a sharp increase in Asn (**Figure 2d**). The diet high in Asn did not affect the capacity of P-ASP to remove Asn from the blood up to 5 days post-injection. Despite not expecting P-ASP to enter the gut, the Asn-diet depleted mouse fecal samples had decreased Asn post-P-ASP, indicating that there could be feedback regulation between the blood and gut. One mouse among the group had increased Asn post-P-ASP, demonstrating that there are unpredictable factors affecting Asn metabolism.

Blood Asn, Gln, and Glu levels were not significantly different between diet groups throughout the study period. Blood aspartic acid levels were significantly higher in the Asn-rich group on day 72 in comparison to the Asn-depleted group (**Figure 2d**. Fecal aspartic acid, glutamine and glutamate levels were not significantly different between groups throughout the study period. Fecal Asn levels were significantly lower in the Asn-rich group on days 72 and 77 in comparison to the Asn-depleted group (**Figure 2e**). Additional metabolites were measured and significance tested between diets (**Supplementary Tables 3** and **4**). The fact that there was no significant difference in Asn levels in the fecal after 35 days on diet indicates that the changes resulting in a difference at day 72 took a while to manifest (**Figure 2e**). Surprisingly, mice on the diet high in Asn had less Asn in the fecal after 72 days which could indicate that the mice may have undergone biological changes to remove excess Asn in the gut. The corresponding increase in blood Asp on day 72 in the Asn rich diet provides one possible explanation.

**Figure 3:**
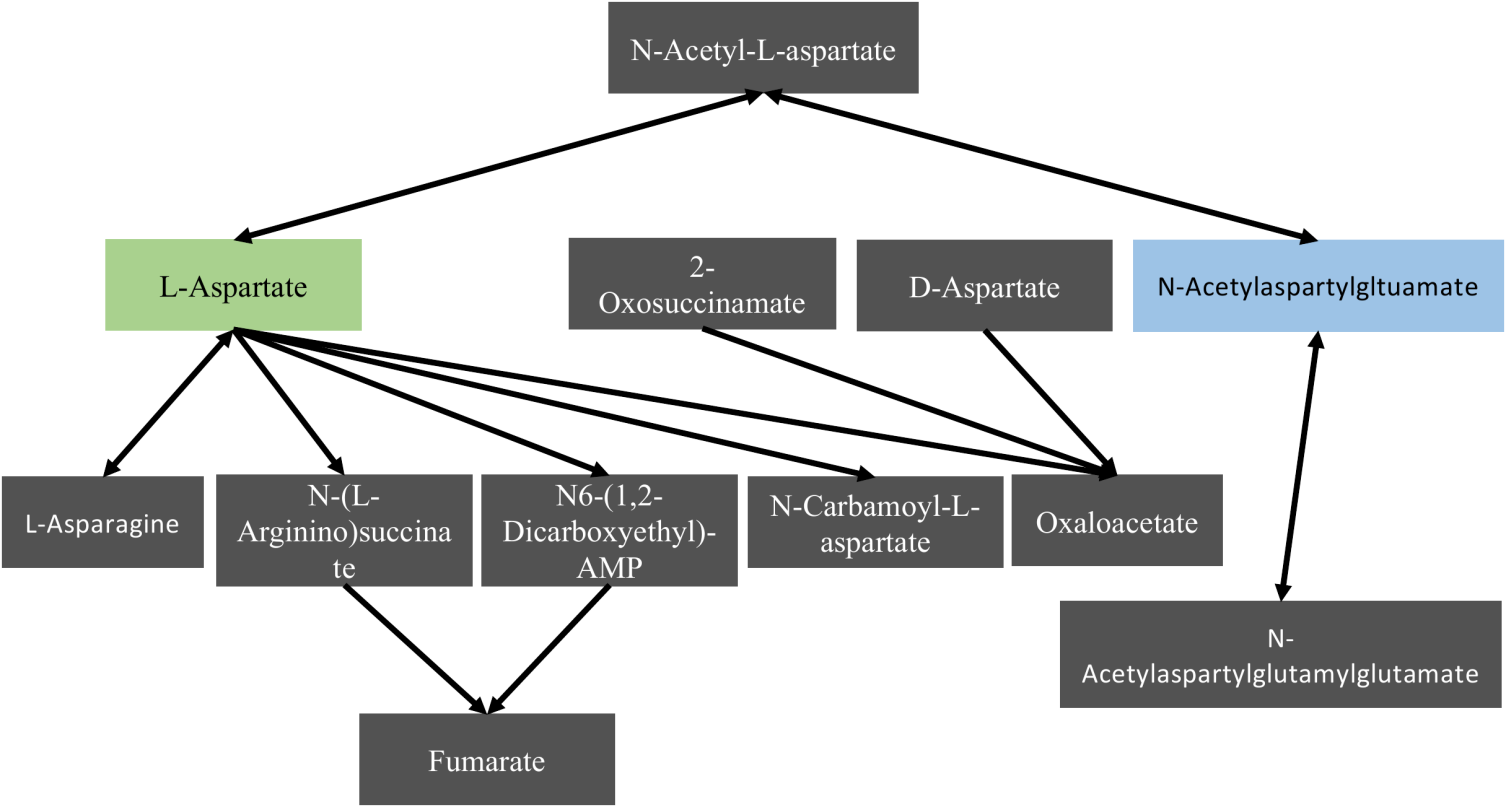
Highlights of the aspartate metabolism pathway. The aspartate metabolism pathway in the blood after 72 days on diet illustrating diet related differences. The green box was significantly higher for the Asn rich diet, and the blue box was significantly higher for the Asn depleted diet.

Metaboanalyst 5.0 was used to identify diet-impacted pathways. There were 5 noteworthy pathways in the blood during pre-diet, 10 on day 35, 12 on day 72, and 4 on day 77 that were significantly different between diets (**Supplementary Table 4**). One pathway of particular interest was on day 72, the alanine, aspartate, and glutamate metabolic pathway, responsible for maintaining metabolic balance. In this pathway, aspartate was significantly higher in the Asn rich group and L-acetylaspartylglutamate was higher in the Asn depleted group (**Figure 3**). Changes in the pathway signify that the mice respond to dietary Asn depletion through either increasing Aspartate or N-acetylaspartylglutamate.

In the feces there were 20 pathways pre-diet, 5 on day 35, 4 on day 72, and 1 on day 77 that were significantly different between diets. Pathways affected in the feces were different from pathways affected in the blood at the same sample time point (**Supplementary Table 5**).

### 3.2 Gut microbiome

**Figure 4:**
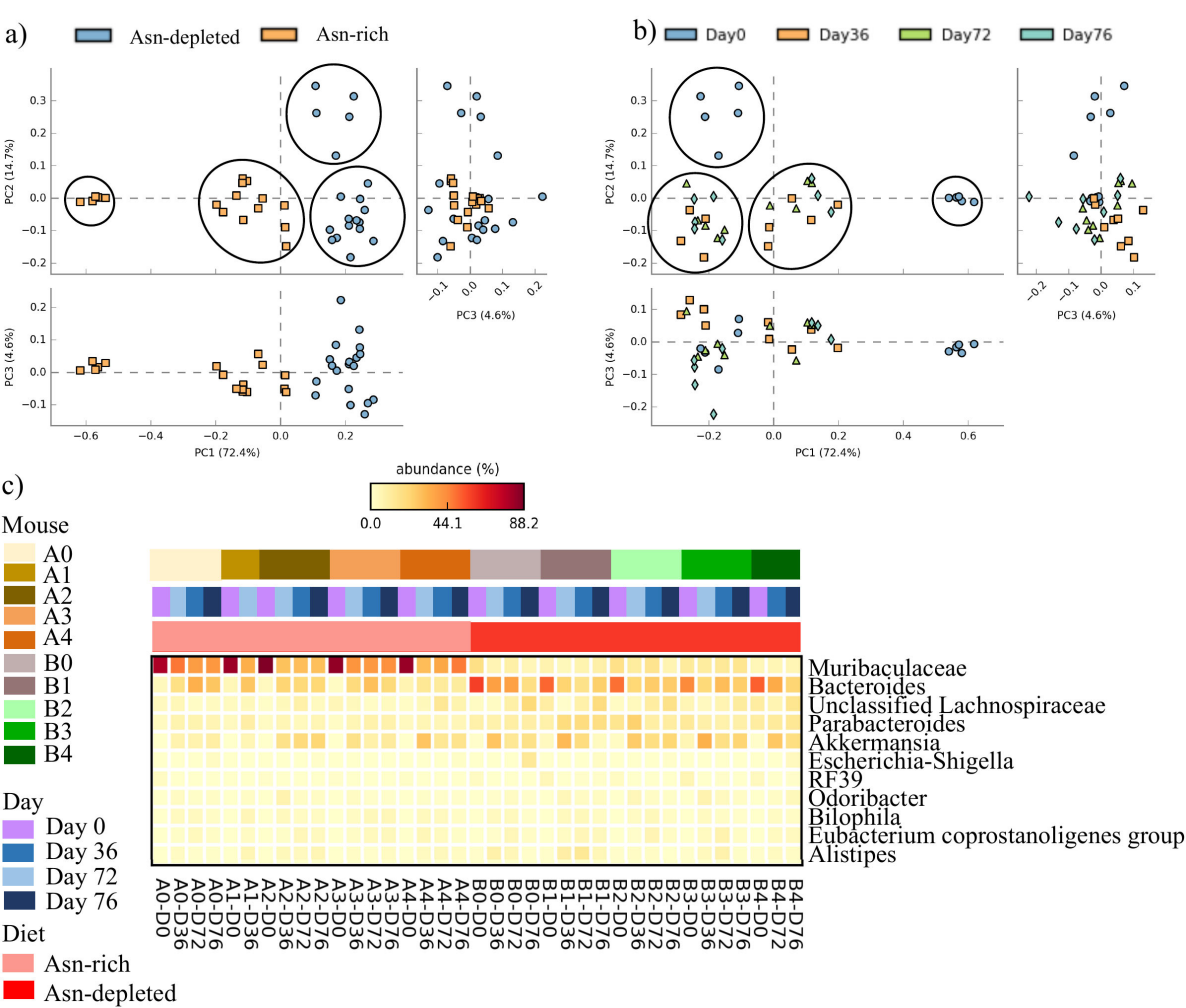
Fecal microbiome results. **(a)** fecal bacteria PCA plot coloured by diet. Samples clustered separately based on diet. **(b)** 16S fecal bacteria PCA plot coloured by day. Samples clustered into four distinct groups, with two clusters for day 0 and two clusters for all day 36, 72, and 76 samples. **(c)** Heatmap at the genus level for bacteria that changed during the study period. The heatmap is colored by diet, day and mouse. Muribaculaceae were in high abundance in the Asn rich treatment group on day 0 but were present in a low abundance in the Asn depleted group. Bacteroides were present in high abundance in the Asn depleted group on day 0 but not in the Asn rich group.

16S sequencing (V4-V5 region) was used to identify the relative abundance of bacteria at multiple taxonomic levels. Microbiome samples were run in duplicate. STAMP was used to analyze sequence data (Parks et al., 2014). PCA plots on the PC1 vs PC2 axis demonstrated four distinct clusters. Samples clustered based on treatment group as well as the day 0 samples clustering separately from the day 36, 72 and 76 samples (**Figures 4a, b**). The PC1 vs PC2 and PC1 vs PC3 plots showed two distinct clusters with the depleted diet on day 0 clustering separately on the PC1 vs PC2 and the rich diet on day 0 clustering separately on the PC2 vs PC3 axis. Surprisingly, the PCA plots demonstrate that the bacterial microbiome was different between the two cages of mice prior to starting the study. Days 36, 72 and 76 clustered into 2 groups based on diet, showing the mice microbiomes remained different across treatment groups yet there was no difference within a diet, post-diet, and post-P-ASP (**Figure 4c**).

A heatmap summarizing data at the genus level shows that mice consuming the Asn rich diet had a high abundance of *Muribaculaceae* prior to starting the study (**Figure 4c**). After 36 days on diet the *Muribaculaceae* abundance was significantly reduced and this decrease persisted through days 72 and 77. The Asn depleted mice had a low starting abundance of *Muribaculaceae* which persisted through the study. The Asn depleted group had a high abundance of *Bacteroides* pre-diet but this significantly declined after being on diet for 36 days and persisted at a low level during the remainder of the study. The Asn rich group started with a low abundance of *Bacteroides* which increased significantly after 36 days on diet. On day 36 the relative increase in *Bacterioides* within the Asn-rich group and decrease within the Asn-depleted group resulted in a similar abundance between groups. Both Asn rich and depleted mice had undetectable *Akkermansia* pre-diet but the bacteria increased in abundance after 36 days in mice on both diets. As the mice remained on diet (day 72) the abundance of *Akkermansia* continued to significantly increase for Asn-depleted mice but maintained a similar level for the Asn-rich mice (**Figure 4c**). The heatmap data show which bacterial genus was responsible for the differences seen between diets at baseline and how they became more similar, through changes in *Muribaculaceae* and *Bacteroides.* Differences in bacteria species within and between groups are presented in **Supplementary Tables 6**-**8**.

Metagenome analysis was run on day 72 samples to determine whether bacterial functional changes could account for the Asp differences between groups. When we compared the groups for asparaginase and asparagine synthetase between diet groups there were no significant differences. Nevertheless, there were 16 bacterial pathways that were significantly different between groups but it is unlikely the changes are directly relevant to Asn metabolism (**Supplementary Table 9**).

## 4 Discussion

P-ASP was added to the pediatric combination chemotherapy regimens based on a relatively simple understanding of the mechanism of action, the catabolism of Asn, with depletion leading to apoptosis of leukemic cells. There are at least three possible sources of blood Asn, absorption through diet, cell synthesis (using asparagine synthetase), and bacteria (which may possess asparaginase and asparagine synthetase). Lowering blood Asn through diet may be an approach to lowering the dose of P-ASP necessary yet there are no human studies addressing this idea. The only study controlling diet was published in 1966, before asparaginase was in use, when Halikowski and co-workers trialed a low-protein low-purine diet in 13 pediatric ALL patients, and all the patients showed an increase in treatment response (8). Asn is rich in animal protein, and we can only speculate that the diet may have contributed to lower blood Asn and the beneficial effect reported in the study. On the other hand, an investigation using mice provided with controlled amounts of Asn in their diets reported that a short-term diet lacking in Asn indeed resulted in low blood Asn (11). The effect of the low Asn diet on cancer metastasis was similar to the effect of multiple injection of L-asparaginase. Also in the Knott et al. study, Asn levels in the blood were significantly higher after 7 days of an Asn rich (4%) diet compared to mice on an Asn depleted diet (11).

Through the present study we sought to determine whether controlled dietary Asn over a longer term can also modify blood Asn levels and also potentially impact P-ASP therapy. We also chose to look for any contribution from gut bacteria in the balance. We anticipated that blood Asn levels would be linked to gut Asn levels, similar to the precedent by Knott et al. in 2018 exploring the impact of a week-long diet with measured Asn on model breast cancer metastasis (11). Mice in our study were maintained on diet for 72 days to determine if prolonged exposure to the diet would be unhealthy, modify blood Asn levels, and lead to adaptive changes in gut bacteria. Contrary to our predictions, on day 35 of the diet the Asn depleted group registered a significant increase in blood Asn, that was followed by the Asn levels returning to baseline by day 72. A significant source of Asn is local production by healthy cells and the animals’ metabolism may have reacted to the absence of dietary Asn with robust synthesis. Such a change may be reflected in asparagine synthetase mRNA and/or protein levels, a measure we did not conduct. Also contrary to our prediction, the Asn rich diet did not result in significantly higher blood Asn levels at either days 35 or 72. Therefore, we conclude that dietary Asn modification may have an acute effect (7 days) as reported by Knott et al. but is less impactful over a prolonged time; the animals’ metabolism appears to reestablish serum levels and the source of Asn remains unclear. This could occur through changes in synthesis, by reduced uptake by intestinal epithelial cells, or both.

Aspartic acid has a close relationship with Asn, being a product of metabolism of Asn by gut bacteria and a substrate for the synthesis of Asn through asparagine synthetase. On day 72, blood Asp levels were significantly higher in the Asn rich diet compared to the Asn depleted diet. As there is no asparaginase activity in mouse (or human) blood, the increased aspartic acid would have to be through a different mechanism. When metagenome analysis was run on the day 72 samples there was no significant difference between groups for bacteria with asparaginase or asparagine synthetase activity. This makes it unlikely that bacterial enzymes are responsible for the increased Asp. One caveat is that we only ran metagenome analysis on day 72 samples, so it remains unclear whether the bacterial asparaginase and asparagine synthetase was changing over time within either group.

Surprisingly, when comparing fecal Asn levels between groups, the Asn rich group had significantly less fecal Asn compared to the depleted diet. This result is the opposite of what we would have predicted, as the diet with high Asn would presumably contribute to higher fecal Asn levels. If we had found a corresponding increase in blood Asn in the rich group, the results may have been due to the gut dumping the excess Asn into the blood, something we did not observe. Considering that Asn did not rise in the blood and there were no changes in bacterial asparagine synthetase or asparaginase, it makes it difficult to predict the mechanism behind this finding. One additional piece of the puzzle could be the increased aspartic acid in the Asn rich diet. This leads to the question: could dietary Asn be replacing any requirement for cells to synthesize Asn, resulting in an accumulation of Asp? Alternatively, despite there being no difference in bacterial asparaginase, could there have been enough asparaginase activity to hydrolyze the Asn in the gut into aspartic acid? Future studies measuring asparagine synthetase activity prior to and during the diet as well as pulse/chase studies where Asn is tracked from the gut into the blood stream may answer this question.

In our study we were able to illustrate how dietary changes and P-ASP can impact the gut bacterial diversity (albeit in feces, not the mucosa) of mice. In 2019, Wang et al. reviewed the published 16S microbiome datasets from mice and identified 37 genera present in most samples, including *Bacteroides, Lachnospiraceae* and *Parabacteroides*, which were also identified in high abundance in our study (20). *Akkermansia* reportedly fell below the threshold in 44.6% of mice but it was a prevalent genus in our study. One genus not included in Wang et al. was *Muribaculaceae*, which was present in high abundance on day 0 in our mice. This may indicate that it may not be a common gut bacterium in mice or speaks to the fact that, not surprisingly, there are strong regional/facilities differences. What was apparent was there was no clear relationship between bacterial enzymes and Asn levels in either the feces or blood, implying there are other confounding influencing the diet/blood Asn relationship. One such confounder is production by eukaryotic cells.

One limitation of the present study is the use of only female mice. There could be sex differences that impact Asn and future research should examine this possibility. Also, none of the mice were modeling ALL by bearing cancer cells, which could impact the animals’ metabolism.

Our study determined that prolonged consumption of diets depleted or rich in Asn, in contrast to a week-long diet, do not impact blood Asn levels. As Asn levels were balanced by day 72, we also did not find any differential impact of dietary Asn on P-ASP efficacy, and P-ASP at the dose we used significantly depleted blood Asn over 5 days with no signs of morbidity. Our protocol to quench P-ASP in blood samples meant that we recovered cellular metabolites in addition to metabolites soluble in the blood, so the depletion by P-ASP was profound. This was also the outcome despite some mice continuing the 4% Asn diet. This outcome may be evidence that there is a feedback system between the gut and serum that helps ensure blood Asn levels are controlled. The P-ASP dose may need to be titrated downward to possibly expose differences in the efficacy of P-ASP to suppress blood Asn in mice on the 2 diets. Ultimately, the optimal dose of P-ASP needs to be determined on leukemic cells in mice, combined with control of dietary Asn. There remains a substantial gap when considering how to apply these findings to benefit patients with ALL. Further research is needed to explore the potential relationship between short-term dietary Asn content in combination with P-ASP.

## Data Availability

The data presented in the study are deposited in the Metabolome Data, MetaboLights: https://www.ebi.ac.uk/metabolights/MTBLS13764 and Microbiome Data, NIH SRA: https://www.ncbi.nlm.nih.gov/sra/PRJNA1401812.
