# Supplementary material for: Investigating diet to control asparagine uptake as an adjunct to asparaginase treatment
Source: Front Oncol. 2026 Jan 30;15:1634113. doi: 10.3389/fonc.2025.1634113 (PMC12900681; doi:10.3389/fonc.2025.1634113)
Supplement: Supplementary file 1 [file Table1.docx]

Supplemental Data

SupplementaryTable 1. Diet components of the asparagine-depleted diet purchased from Dyets.


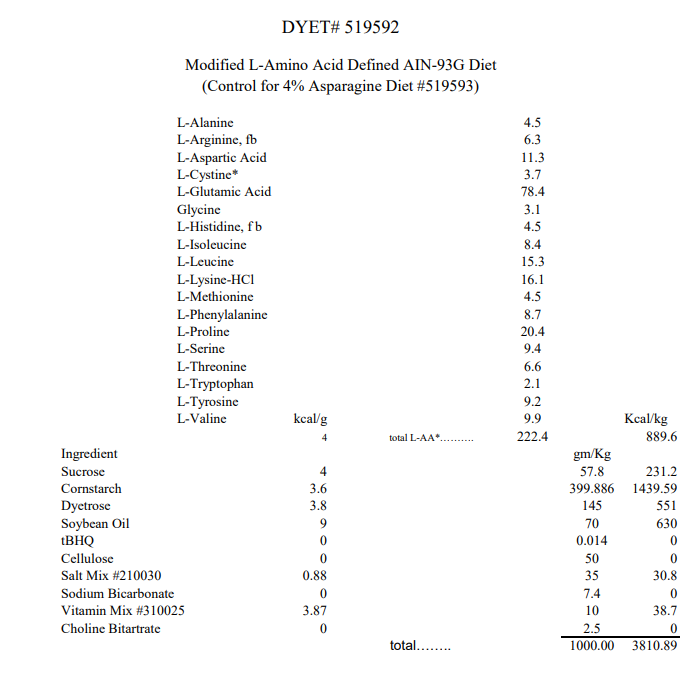


Supplementary Table 2. Diet components of the asparagine-rich diet purchased from Dyets.


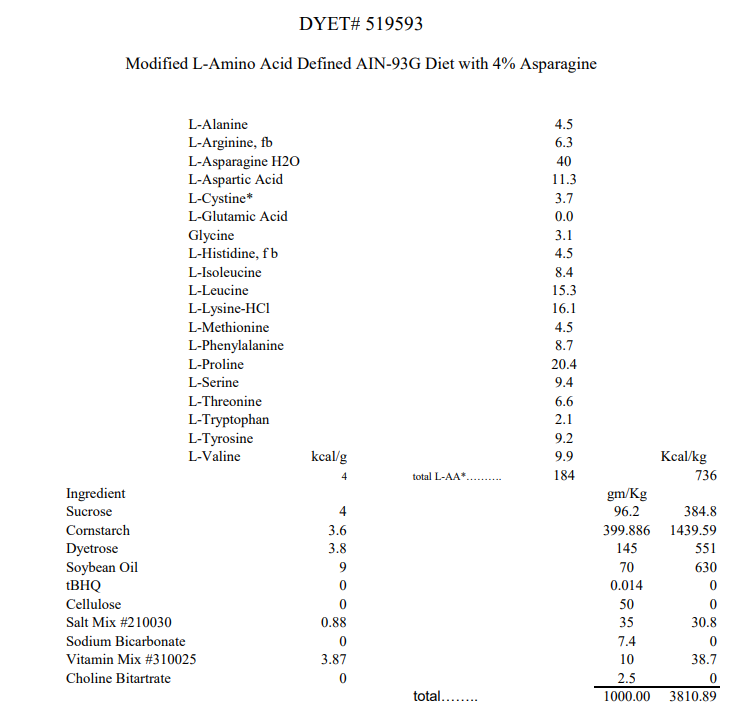


Supplementary Table 3. Blood metabolites that were significantly different between diets, determined using ANCOVA. Day 35 and day 72 results used day 0 as the control. Day 77 (e.g. 5 days post P-ASP injection) results used Day 72 (prior to P-ASP injection) as the control. * marks significance p<0.05.

| **Metabolite** | **Day 0 to 35** | **Day 0 to 72** | **Day 72 to 77** |
| --- | --- | --- | --- |
| Adenosine |  | ***** |  |
| Arachidonyl Carnitine | ***** |  |  |
| Betaine | ***** |  |  |
| Choline | ***** |  |  |
| Cinnamoylglycine |  | ***** |  |
| Creatine | ***** |  |  |
| Creatinine | ***** |  |  |
| Decanolycarnitine | ***** |  |  |
| 9-Decenoylcarnitine | ***** |  |  |
| Dodecanoylcarnitine | ***** |  |  |
| Glucaric Acid | ***** |  |  |
| Glutarylcarnitine | ***** |  |  |
| 8-HETE |  | ***** | ***** |
| Hydroxyhexanoycarnitine | ***** |  |  |
| 5-Hydroxyindoleacetic acid | ***** |  |  |
| Imidazoleacetic Acid | ***** |  |  |
| ITP |  |  | ***** |
| 2-Keto-L-gluconate | ***** |  |  |
| L-Aspartic Acid |  | ***** |  |
| L-2-hydroxyglutaric Acid | ***** | ***** |  |
| Linoleyl carnitine | ***** |  |  |
| L-Octanoylcarnitine | ***** |  |  |
| L-phenylalanine |  |  | ***** |
| L-Tryptophan | ***** |  |  |
| Malonic Acid | ***** |  |  |
| Metanephrine |  |  | ***** |
| Methylguanidine | ***** |  |  |
| 7-Methylguanine |  | ***** |  |
| N6-Acetyl-L-Lysine | ***** |  |  |
| N-Acetyl-D-glucosamine |  | ***** |  |
| Nicotinamide ribotide |  |  | ***** |
| O-acetylserine |  |  | ***** |
| 2-Octenoylcarnitine |  |  | ***** |
| Oleoylcarnitine |  |  | ***** |
| Ortho-hydrxyphenylacetic acid | ***** |  |  |
| Propionylcarnitine | ***** |  |  |
| Pyridoxal |  |  | ***** |
| 4-Pyridoxic Acid | ***** | ***** |  |
| Quinolinic Acid |  |  | ***** |
| Sphingosine |  |  | ***** |
| Stearoylcarnitine | ***** | ***** |  |
| Sucrose |  | ***** |  |
| Tetradecadiencarnitine | ***** |  |  |
| Tetradecanoylcarnitine | ***** |  |  |
| Thiamine |  |  | ***** |
| Thymidine |  | ***** | ***** |
| Trans-2-dodecenoylcarnitine | ***** |  |  |
| Tryptamine |  | ***** | ***** |
| Uracil |  |  | ***** |

Supplementary Table 3. Stool metabolites that were significantly different between diets, determined by ANCOVA. Day 35 and day 72 results used day 0 as the control. Day 77 results used day 72 as the control. * marks significance p<0.05.

| **Metabolite** | **Day 35** | **Day 72** | **Day 77** |
| --- | --- | --- | --- |
| **Adenine** | ***** | ***** |  |
| **Adipic acid** | ***** |  |  |
| **ADP** |  | ***** |  |
| **2-Arachidonylgylcerol** |  |  | ***** |
| **Beta-Alanine** | ***** | ***** |  |
| **Betaine** |  |  | ***** |
| **Cyclophosphamide** |  |  | ***** |
| **Cytidine** |  |  | ***** |
| **Cytosine** | ***** | ***** |  |
| **dADP** | ***** |  |  |
| **Deoxyuridine** | ***** |  |  |
| **dGDP** | ***** |  |  |
| **Dopamine** | ***** |  |  |
| **dTDP** |  | ***** |  |
| **Epinephrine** | ***** |  | ***** |
| **11,12-Epoxyeicosatrienoic acid** | ***** |  |  |
| **Hypotaurine** | ***** |  |  |
| **L-Asparagine** |  | ***** | ***** |
| **L-Cysteine** |  | ***** |  |
| **L-Homocysteic acid** | ***** | ***** |  |
| **Linoleic acid** | ***** |  |  |
| **L-Isoleucine** | ***** |  |  |
| **L-Leucine** |  | ***** |  |
| **3-Methylindole** |  | ***** |  |
| **N-Acetyl-D-glucosamine** | ***** |  |  |
| **N-Acetyl-L-alanine** | ***** |  |  |
| **Normetanephrine** |  | ***** |  |
| **Ornithine** | ***** |  |  |
| **8,9-DiHETrE** | ***** |  |  |
| **Pantothenic Acid** | ***** |  |  |
| **Phenylacetlyglutamine** |  | ***** |  |
| **Picolinic acid** | ***** |  | ***** |
| **Pipecolic acid** | ***** |  |  |
| **Prostaglandin F2a** | ***** |  |  |
| **S-Adenosylmethioninamine** |  |  | ***** |
| **Sphinganine** |  | ***** |  |
| **tetradecanoylcarnitine** | ***** |  |  |
| **tetrahydrobiopterin** | ***** |  |  |
| **Tiglyglycine** | ***** |  |  |
| **Tyramine** |  |  | ***** |
| **Uracil** | ***** |  |  |
| **Uridine 5'-monophosphate** |  |  | ***** |

Supplementary Table 4. Pathway blood analysis results from Metaboanalyst. P values listed for significant pathways by timepoint.

| **Pathway Affected** | **Day 0** | **Day 35** | **Day 72** | **Day 77** |
| --- | --- | --- | --- | --- |
| Alanine, aspartate and glutamate metabolism |  |  | 0.0192 |  |
| Arginine biosynthesis |  |  | 0.046 |  |
| Biotin metabolism |  | 0.0241 |  |  |
| Citrate cycle (TCA cycle) |  | 0.0287 |  |  |
| Glycerolipid metabolism |  |  | 0.00543 |  |
| Glycerophospholipid metabolism |  |  | 0.00566 |  |
| Glycine, serine, and threonine metabolism |  | 0.021 |  |  |
| Glycosylphosphatidylinositol (GPI)- anchor biosynthesis | 0.0456 |  | 0.00641 |  |
| Glyoxylate and dicarboxylate metabolism |  | 0.0387 |  |  |
| Lysine degradation |  | 0.0155 |  |  |
| Mannose type O-glycan biosynthesis | 0.0269 |  |  |  |
| Nicotinate and nicotinamide metabolism |  |  | 0.0438 | 0.045646 |
| Pantothenate and CoA biosynthesis |  |  | 0.0315 |  |
| Phenylalanine metabolism |  |  | 0.0488 | 0.012624 |
| Phenylalanine, tyrosine and tryptophan biosynthesis |  |  |  | 0.015935 |
| Propanoate metabolism |  | 0.0267 |  |  |
| pyrimidine metabolism |  |  |  |  |
| Retinol metabolism |  |  | 0.0431 |  |
| Sphingolipid metabolism |  |  |  |  |
| Starch and sucrose metabolism |  |  | 0.0296 |  |
| Thiamine metabolism |  | 0.016 |  | 0.01714 |
| Tryptophan metabolism |  | 0.0176 | 0.016 |  |
| Ubiquinone and other terpenoid-quinone biosynthesis | 0.0438 |  |  |  |
| Valine, leucine and isoleucine degradation | 0.0139 | 0.0257 |  |  |
| Valine, Leucine, and isoleucine biosynthesis | 0.011 |  |  |  |
| Vitamin B6 metabolism |  | 0.0312 | 0.0169 |  |

Supplementary Table 5. Pathway stool analysis results from Metaboanalyst. P values listed for significant pathways by timepoint.

| **Pathway Affected** | **Day 0** | **Day 35** | **Day 72** | **Day 77** |
| --- | --- | --- | --- | --- |
| Arachidonic acid metabolism | 0.041 |  |  |  |
| Ascorbate and aldarate metabolism |  | 0.03 |  |  |
| Biosynthesis of unsaturated fatty acids |  | 0.001 |  |  |
| Cysteine and methionine metabolism | 0.002 |  |  |  |
| Folate biosynthesis |  | 2.38E-04 |  |  |
| Glutathione metabolism | 0.007 |  |  |  |
| Glycerophospholipid metabolism | 0.020 |  |  |  |
| Glycine, serine and threonine metabolism | 0.021 |  |  |  |
| Inositol phosphate metabolism | 0.016 |  |  |  |
| Linoleic acid metabolism |  | 0.003 |  |  |
| Mannose type O-glycan biosynthesis | 0.007 |  |  |  |
| Neomycin, kanamycin and gentamicin biosynthesis | 0.016 |  |  |  |
| Nicotinate and nicotinamide metabolism | 0.001 |  |  |  |
| Phenylalanine metabolism | 0.037 |  |  |  |
| Phenylalanine, tyrosine and tryptophan biosynthesis | 0.037 |  |  |  |
| Phosphatidylinositol signaling system | 0.003 |  |  |  |
| Propanoate metabolism |  |  | 0.014 | 0.020 |
| Purine metabolism | 0.007 |  |  |  |
| Pyrimidine metabolism |  |  | 0.041 |  |
| Retinol metabolism | 0.025 |  |  |  |
| Starch and sucrose metabolism | 0.017 |  |  |  |
| Taurine and hypotaurine metabolism | 0.001 |  |  |  |
| Terpenoid backbone biosynthesis |  |  | 0.040 |  |
| Thiamine metabolism | 0.006 |  |  |  |
| Tryptophan metabolism | 0.032 |  |  |  |
| Tyrosine metabolism | 0.005 | 0.003 |  |  |
| Ubiquinone and other terpenoid-quinone biosynthesis | 0.038 |  |  |  |

Supplementary Table 6. Bacteria that were significantly different between treatment groups pre-diet (day 0), after 72 days on diet, and 5 days post-P-ASP (day 77). The diet with the higher abundance of the bacteria is reported in the table. Greyed boxes were not significantly different between diets. A p value ≤0.05 was considered significant.

| **Species** | **Day 0** | **Day 36** | **Day 72** | **Day 77** |
| --- | --- | --- | --- | --- |
| *Alistipes finegoldii* | Depleted |  |  |  |
| *Alistipes obesi* | Depleted |  |  |  |
| *Alistipes shahii* | Depleted |  |  |  |
| *Bacteroides caccae* | Depleted |  |  |  |
| *Bacteroides dorei* | Depleted |  |  | Rich |
| *Bacteroides fragilis* | Depleted |  |  |  |
| *Bacteroides vulgatus* | Depleted |  | Depleted | Depleted |
| *Clostridiales bacterium* |  |  |  | Depleted |
| *Clostridium fusiformis* |  |  |  | Depleted |
| *Clostridium leptum* |  |  | Depleted | Depleted |
| *Lachnospiraceae bacterium* |  |  |  | Depleted |
| *Lactobacillus murinus* |  |  | Rich |  |
| *Mouse gut* | Depleted |  |  |  |
| *Parabacteroides distasonis* | Depleted |  |  | Depleted |
| *Parabacteroides goldsteinii* | Depleted |  |  |  |
| *Parabacteroides merdae* | Depleted |  |  |  |
| Unclassified Acetatifactor |  |  |  | Depleted |
| Unclassified *Acetivibrio ethanolgignen*s |  |  |  | Depleted |
| Unclassified Adlercreutzia | Depleted | Rich | Rich |  |
| Unclassified Akkermansia |  | Depleted |  |  |
| Unclassified Alistipes |  |  |  | Rich |
| Unclassified Anaerotruncus |  |  |  | Depleted |
| Unclassified Bacteroides | Depleted | Depleted | Depleted | Depleted |
| Unclassified Christensenellaceae R-7 |  |  |  | Depleted |
| Unclassified Clostridia UCG-014 | Depleted |  |  |  |
| Unclassified Clostridia vadinBB60 | Depleted |  |  |  |
| Unclassified Clostridium sensu stricto | Depleted | Depleted | Depleted | Depleted |
| Unclassified Erysipelatoclostridiaceae |  |  |  | Depleted |
| Unclassified Erysipelatoclostridium | Depleted |  |  | Depleted |
| Unclassified GCA-900066575 | Depleted |  |  | Depleted |
| Unclassified Lachnospiraceae |  | Rich |  | Depleted |
| Unclassified Lactobacillus |  |  |  | Rich |
| Unclassified Muribaculaceae | Rich | Rich | Rich | Rich |
| Unclassified Negativibacillus |  | Rich |  |  |
| Unclassified Odoribacter |  |  | Depleted |  |
| Unclassified Oscillospirales | Depleted | Rich |  |  |
| Unclassified Parabacteroides | Depleted |  | Depleted | Depleted |
| Unclassified Paraprevotella | Depleted |  |  | Depleted |
| Unclassified Parasutterella | Depleted |  |  |  |
| Unclassified Peptococcaceae | Depleted |  |  | Depleted |
| Unclassified RF39 | Depleted |  |  |  |
| Unclassified Rhodospirillales |  | Depleted |  |  |
| Unclassified Romboutsia | Depleted |  | Depleted | Depleted |
| Unclassified Ruminococcaceae |  |  |  | Depleted |
| Unclassified Ruminococcus | Depleted |  |  |  |
| Unclassified Turicibacter | Depleted |  | Depleted |  |
| Unclassified Tuzzerella |  |  |  | Depleted |
| Unclassified UCG-005 | Depleted |  |  |  |

Supplementary Table 7. Differences in bacteria within a group for the Asn-rich diet when comparing pre-diet (day 0) to 72 days on diet and 72 days on diet to 4 days post P-ASP (day 76). Only significant results (p≤0.05) are shown and the day with the higher abundance of bacteria is indicated.

| **Name** | **Day 0-36** | **Day 0-72** | **Day 36-72** | **Day 72-76** |
| --- | --- | --- | --- | --- |
| *Alistipes finegoldii* | Day 0 |  |  |  |
| *Alistipes onderdonkii* | Day 36 |  |  |  |
| *Alistipes shahii* | Day 0 | Day 0 |  |  |
| *Bacteroides caccae* | Day 36 | Day 72 |  |  |
| *Bacteroides dorei* |  | Day 72 | Day 72 |  |
| *Bacteroides fragilis* | Day 36 |  |  |  |
| *Bacteroides vulgatus* | Day 36 | Day 72 | Day 36 |  |
| *Blautia coccoides* | Day 0 | Day 0 |  |  |
| *Lachnospiraceae* | Day 0 | Day 0 |  |  |
| *Lachnospiraceae bacterium* | Day 36 |  |  |  |
| *Lactobacillus murinus* |  | Day 72 |  |  |
| *Parabacteroides goldsteinii* | Day 0 |  |  |  |
| Unclassified Adlercreutzia |  | Day 72 | Day 72 | Day 72 |
| Unclassified Akkermansia | Day 36 | Day 72 |  |  |
| Unclassified Alcaligenes | Day 0 |  |  |  |
| Unclassified Bilophila | Day 36 | Day 72 |  |  |
| Unclassified Clostridia vadinBB60 | Day 0 |  |  |  |
| Unclassified Clostridium sensu stricto | Day 0 |  |  |  |
| Unclassified Colidextribacter | Day 36 |  |  |  |
| Unclassified Erysipelatoclostridium |  |  |  | Day 72 |
| Unclassified *Eubacterium coprostanoligenes* | Day 36 | Day 72 |  |  |
| Unclassified Intestinimonas | Day 0 |  |  |  |
| Unclassified Lachnospiraceae NK4A136 | Day 0 |  |  |  |
| Unclassified Lachnospiraceae UCG-001 | Day 0 | Day 0 |  |  |
| Unclassified Lactobacillus |  |  | Day 72 |  |
| Unclassified Muribaculaceae | Day 0 | Day 0 |  |  |
| Unclassified Odoribacter | Day 36 | Day 72 |  |  |
| Unclassified Oscillibacter | Day 36 |  | Day 36 |  |
| Unclassified Parabacteroides | Day 36 | Day 72 |  |  |
| Unclassified RF39 | Day 0 |  |  |  |
| Unclassified Rhodospirillales | Day 36 |  |  |  |
| Unclassified Romboutsia | Day 36 |  | Day 36 |  |
| Unclassified Roseburia | Day 36 |  |  |  |
| Unclassified Ruminococcaceae | Day 36 |  |  |  |
| Unclassified Turicibacter | Day 0 | Day 0 |  |  |
| Unclassified Tyzzerella | Day 0 | Day 0 |  |  |

Supplementary Table 8. Differences in bacteria within a group for the Asn-depleted diet when comparing pre-diet (day 0) to 72 days on diet and 72 days on diet to 4 days post P-ASP (day 76). Only significant results (p≤0.05) are shown and the day with the higher abundance of bacteria is indicated.

| **Name** | **Day 0-36** | **Day 0-72** | **Day 36-72** | **Day 72-76** |
| --- | --- | --- | --- | --- |
| *Alistipes finegoldii* | Day 0 | Day 0 |  | Day 76 |
| *Alistipes obesi* | Day 0 | Day 0 |  |  |
| *Alistipes onderdonkii* | Day 36 | Day 72 |  |  |
| *Alistipes shahii* | Day 0 | Day 0 |  |  |
| *Bacteroides dorei* | Day 0 | Day 0 |  |  |
| *Bacteroides fragilis* | Day 36 |  |  |  |
| *Blautia coccoides* | Day 0 | Day 0 |  |  |
| *Clostridiales bacterium* |  |  |  | Day 76 |
| *Clostridium* |  |  |  | Day 76 |
| *Clostridium leptum* |  | Day 72 |  |  |
| *Clostridiumfusiformis* |  |  |  | Day 76 |
| *Lachnospiraceae bacterium* | Day 36 | Day 72 |  |  |
| Mouse gut | Day 0 | Day 0 |  |  |
| Parabacteroides goldsteinii | Day 0 |  |  |  |
| Parabacteroides merdae | Day 0 | Day 0 | Day 72 |  |
| Unclassified Acetatifactor |  |  |  | Day 76 |
| Unclassified Acetivibrio ethanolgignens |  | Day 72 |  | Day 76 |
| Unclassified Adlercreutzia | Day 0 |  |  |  |
| Unclassified Akkermansia | Day 36 | Day 72 | Day 36 |  |
| Unclassified Alcaligenes | Day 0 | Day 0 |  |  |
| Unclassified Bacteroides | Day 0 | Day 0 |  |  |
| Unclassified Bilophila | Day 36 | Day 72 |  | Day 72 |
| Unclassified Clostridia UCG-014 | Day 0 | Day 0 |  |  |
| Unclassified Clostridia vadinBB60 | Day 0 | Day 0 |  |  |
| Unclassified Clostridium sensu stricto |  | Day 0 |  |  |
| Unclassified Colidextribacter |  |  |  | Day 76 |
| Unclassified Erysipelatoclostridium |  | Day 72 | Day 72 |  |
| Unclassified Eubacterium coprostanoligenes | Day 36 | Day 72 | Day 72 | Day 72 |
| Unclassified Eubacterium siraeum | Day 0 | Day 0 |  |  |
| Unclassified GCA-900066575 |  | Day 72 |  | Day 76 |
| Unclassified Lachnospiraceae | Day 0 |  | Day 72 | Day 76 |
| Unclassified Lachnospiraceae NK4A136 |  | Day 0 |  |  |
| Unclassified Lactobacillus | Day 0 | Day 0 | Day 72 |  |
| Unclassified Negativibacillus | Day 0 |  |  |  |
| Unclassified Odoribacter | Day 36 | Day 72 |  |  |
| Unclassified Oscillibacter |  | Day 72 |  |  |
| Unclassified Oscillospiraceae |  |  |  | Day 76 |
| Unclassified Oscillospirales | Day 0 | Day 0 |  |  |
| Unclassified Parabacteroides | Day 36 | Day 72 |  |  |
| Unclassified Paraprevotella |  | Day 0 |  | Day 76 |
| Unclassified Parasutterella | Day 0 | Day 0 | Day 36 |  |
| Unclassified Peptococcaceae | Day 0 | Day 0 |  | Day 76 |
| Unclassified RF39 | Day 0 | Day 0 |  |  |
| Unclassified Rhodospirillales | Day 36 |  | Day 36 |  |
| Unclassified Romboutsia |  | Day 72 |  |  |
| Unclassified Ruminococcaceae |  | Day 72 |  | Day 76 |
| Unclassified Ruminococcus | Day 0 | Day 0 |  |  |
| Unclassified Turicibacter | Day 0 | Day 0 |  |  |
| Unclassified Tuzzerella |  |  |  | Day 76 |

Supplementary Table 9. Metagenome results for bacterial functions that were significantly different between groups. P value <0.05 was taken for significance and the diet with significantly more bacteria with the specified function is listed in the table.

| **Name** | **P value** | **Higher** |
| --- | --- | --- |
| EC:3.2.1.89\|Arabinogalactan endo-beta-1,4-galactanase | 1.45E-07 | rich |
| EC:3.5.1.5\|Urease | 5.60E-05 | rich |
| EC:4.2.1.130\|D-lactate dehydratase | 1.02E-04 | rich |
| EC:4.1.2.9\|Phosphoketolase | 2.26E-04 | rich |
| EC:3.4.21.26\|Prolyl oligopeptidase | 2.40E-04 | depleted |
| EC:4.1.3.3\|N-acetylneuraminate lyase | 1.43E-03 | rich |
| EC:4.2.1.59\|3-hydroxyacyl-[acyl-carrier-protein] dehydratase | 1.96E-03 | rich |
| EC:2.7.1.82\|Ethanolamine kinase | 4.12E-03 | depleted |
| EC:5.1.1.1\|Alanine racemase | 4.55E-03 | rich |
| EC:2.7.11.32\|not_found | 5.48E-03 | rich |
| EC:2.7.9.1\|Pyruvate, phosphate dikinase | 6.15E-03 | rich |
| EC:6.3.4.5\|Argininosuccinate synthase | 6.51E-03 | rich |
| EC:1.1.1.264\|L-idonate 5-dehydrogenase (NAD(P)(+)) | 7.14E-03 | rich |
| EC:5.3.1.6\|Ribose-5-phosphate isomerase | 7.47E-03 | rich |
| EC:2.3.1.157\|Glucosamine-1-phosphate N-acetyltransferase | 8.46E-03 | rich |
| EC:2.4.1.313\|not_found | 9.95E-03 | depleted |
| EC:3.6.1.7\|Acylphosphatase | 1.30E-02 | rich |
| EC:2.7.7.72\|CCA tRNA nucleotidyltransferase | 0.013 | rich |
| EC:2.7.7.56\|tRNA nucleotidyltransferase | 0.015 | rich |
| EC:2.4.1.7\|Sucrose phosphorylase | 0.016 | rich |
| EC:4.1.1.101\|not_found | 0.016 | rich |
| EC:2.7.1.199\|not_found | 0.017 | rich |
| EC:1.12.1.3\|Hydrogen dehydrogenase (NADP(+)) | 0.018 | depleted |
| EC:1.8.5.3\|Dimethylsulfoxide reductase | 0.018 | rich |
| EC:5.6.2.2\|not_found | 0.018 | rich |
| EC:3.4.14.11\|Xaa-Pro dipeptidyl-peptidase | 0.019 | rich |
| EC:1.5.1.3\|Dihydrofolate reductase | 0.02 | depleted |
| EC:3.5.1.24\|Choloylglycine hydrolase | 0.02 | rich |
| EC:4.2.1.77\|Trans-L-3-hydroxyproline dehydratase | 0.02 | rich |
| EC:2.1.1.198\|16S rRNA (cytidine(1402)-2'-O)-methyltransferase | 0.021 | depleted |
| EC:2.7.7.n1\|not_found | 0.021 | depleted |
| EC:4.4.1.15\|D-cysteine desulfhydrase | 0.022 | depleted |
| EC:2.4.2.6\|Nucleoside deoxyribosyltransferase | 0.023 | rich |
| EC:1.1.1.25\|Shikimate dehydrogenase | 0.024 | rich |
| EC:2.7.1.176\|UDP-N-acetylglucosamine kinase | 0.025 | depleted |
| EC:3.4.22.16\|Cathepsin H | 0.026 | depleted |
| EC:6.3.5.3\|Phosphoribosylformylglycinamidine synthase | 0.026 | rich |
| EC:3.1.26.5\|Ribonuclease P | 0.028 | rich |
| EC:2.1.1.148\|Thymidylate synthase (FAD) | 0.029 | rich |
| EC:2.3.1.89\|Tetrahydrodipicolinate N-acetyltransferase | 0.03 | rich |
| EC:3.2.1.122\|Maltose-6'-phosphate glucosidase | 0.034 | rich |
| EC:3.2.1.151\|Xyloglucan-specific endo-beta-1,4-glucanase | 0.035 | depleted |
| EC:1.1.1.22\|UDP-glucose 6-dehydrogenase | 0.035 | rich |
| EC:2.1.3.15\|not_found | 0.036 | rich |
| EC:2.7.1.16\|Ribulokinase | 0.036 | depleted |
| EC:1.8.4.11\|Peptide-methionine (S)-S-oxide reductase | 0.039 | rich |
| EC:3.2.2.23\|DNA-formamidopyrimidine glycosylase | 0.039 | rich |
| EC:5.1.3.9\|N-acylglucosamine-6-phosphate 2-epimerase | 0.039 | rich |
| EC:1.1.1.27\|L-lactate dehydrogenase | 0.04 | rich |
| EC:3.5.4.13\|dCTP deaminase | 0.04 | depleted |
| EC:6.2.1.54\|not_found | 0.04 | rich |
| EC:5.1.3.2\|UDP-glucose 4-epimerase | 0.041 | rich |
| EC:1.1.5.3\|Glycerol-3-phosphate dehydrogenase | 0.042 | depleted |
| EC:1.3.98.1\|Dihydroorotate oxidase (fumarate) | 0.043 | rich |
| EC:4.2.1.75\|Uroporphyrinogen-III synthase | 0.043 | depleted |
| EC:2.6.1.2\|Alanine transaminase | 0.046 | rich |
| EC:1.7.99.1\|Hydroxylamine reductase | 0.047 | rich |
